# Production of Pharmaceutical Proteins in *Solanaceae* Food Crops

**DOI:** 10.3390/ijms14022753

**Published:** 2013-01-29

**Authors:** Maria Manuela Rigano, Giorgio De Guzman, Amanda M. Walmsley, Luigi Frusciante, Amalia Barone

**Affiliations:** 1 Department of Agricultural Sciences, University of Naples “Federico II”, Via Università 100, Portici 80055, Naples, Italy; E-Mails: mrigano@unina.it (M.M.R.); fruscian@unina.it (L.F.); 2 School of Biological Sciences, Monash University, Clayton, Victoria 3800, Australia; E-Mails: giorgio.deguzman@monash.edu (G.D.G.); amanda.walmsley@monash.edu (A.M.W.)

**Keywords:** plant-made vaccines, *Solanaceae*, transgenic plant, food crop

## Abstract

The benefits of increased safety and cost-effectiveness make vegetable crops appropriate systems for the production and delivery of pharmaceutical proteins. In particular, *Solanaceae* edible crops could be inexpensive biofactories for oral vaccines and other pharmaceutical proteins that can be ingested as minimally processed extracts or as partially purified products. The field of crop plant biotechnology is advancing rapidly due to novel developments in genetic and genomic tools being made available today for the scientific community. In this review, we briefly summarize data now available regarding genomic resources for the *Solanaceae* family. In addition, we describe novel strategies developed for the expression of foreign proteins in vegetable crops and the utilization of these techniques to manufacture pharmaceutical proteins.

## 1. Introduction

Over 3000 species belong to the family *Solanaceae*, which is the third most important plant family (exceeded only by grasses and legumes). It comprises a number of economically important crops, such as tomato, potato, pepper and eggplant [1]. Potato (*Solanum tuberosum*) is the fourth crop in the world in terms of production after wheat, rice and corn. It is important in the human diet since it contains high nutritive value, including quality proteins, mineral salts, starch and vitamins. Tomato (*Solanum lycopersicum*) also largely contributes to dietary nutrition worldwide with beneficial effects mainly attributed to antioxidant compounds in the fruit. In addition, recently, significant progress has been made to improve the levels of human health-promoting compounds (such as carotenoids and anthocyanins) in tomato fruits through metabolic engineering or breeding [2]. Tomato is also ranked as one of the most important vegetable crops in the world with by-products including fresh market fruit and processed paste, juice, sauce and powder.

Due to the economical importance of the *Solanaceae* family and to the genetic and genomic resources available, numerous studies have focused on species belonging to this family. In particular, genetic and genomic studies have focused on potato and tomato since, and unlike other model species such as *Arabidopsis thaliana* and *Nicotiana tabacum*, they produce marketable organs (fruit for tomato and tuber for potato). They have the same basic chromosome number (2*n* = 24) and share similar chromosome architecture, only differing by six inversions [3]. Both exhibit a relatively small genome size (840 and 950 Mb, respectively, as reported by [4]), encoding approximately 35,000 genes that are sequestered in contiguous distal euchromatic regions. In addition, both species have a short generation time and are well-adapted to *in vitro* culture and transformation protocols. All these features have led to rapid and successful development of many genetic and genomics tools, contributing to the improvement in performance of these two food crops. These include molecular marker collections, different mapping populations, BAC libraries, physical maps and mutant populations (available from the Solgenomics network, [5]). Recently, genotyping panels have also been developed for single nucleotide polymorphism (SNP) used for intra- and inter-varietal delineations by the *Solanaceae* Coordinated Agricultural Project of USDA [6]. An increasing amount of ESTs from different tissues and developmental stages have been collected in the SolEST database [7] thus providing a considerable resource for research into gene expression, gene discovery, and comparative genomics studies. Among these, various transcriptomic platforms were also designed for tomato and potato (TIGR *Solanaceae* Genomics Resource, [8]), as well as RNA-seq data were derived by many experiments in both species [9].

More recently, the potato and tomato genomes were sequenced using a combination of “BAC by BAC” and whole genome shotgun sequencing, and by exploiting different next generation sequencing technologies [4,10]. The release of the tomato and potato genome sequences and the continuous development of genetic and genomic resources would enable the development of high-yielding and nutritionally improved varieties by traditional and molecular breeding and by genetic transformation [11].

The engineering of *Solanaceae* food crops by genetic modification have been generally used in order to improve the quality of these crops. Recently, *Solanaceae* crops have been proposed also as advantageous platforms for the production of oral vaccines and other pharmaceutical proteins since they are palatable uncooked, unprocessed or partially processed, and contain orally-active health promoting metabolites [12,13]. In particular, tomato and potato plants have been used as vehicles for the expression and oral delivery of vaccines because they offer unique advantages such as short growth cycle, high nutritional value and abundant biomass at low cost [14–17].

Plant-made pharmaceuticals have gained interest because of their advantages; such as, low production costs, product safety, higher scale-up capacity, lack of risk of contamination with human or animal pathogens, reduction of downstream processing and ability of the plant cells to perform complex post-translational modifications [18,19]. Several plant species (dicot, monocot, food and non-food, leafy crops, cereals and legumes) have been used for the expression of recombinant proteins. Transient expression systems and contained systems based on cultured plant cells or aquatic plants have been adopted [20]. However, in view of the rapidly growing interest in the *Solanaceae* family, this review will summarize the recent progress in producing vaccines and other pharmaceutical proteins in food crops belonging to this important family.

## 2. Available Molecular Tools for Protein Production in *Solanaceae* Crops

While the amplifying deconstructed viral vector transient transformation platform [21] has not yet been effectively demonstrated in *Solanaceae* food crops, several techniques have been used for the production of recombinant protein in these crops including alternative transformation methods such as floral dip or alternative bacteria species [22,23] and an agro-infiltration-based system that allows the transient expression of transgenes directly into tomato fruit tissues [24]. However, integration of the DNA encoding the transgene into the nuclear plant genome by *Agrobacterium*-mediated transformation remains the main process employed, due to its simplicity and cost-effectiveness. In the last 20 years several *Agrobacterium*-mediated transformation protocols have been developed in food crops using cotyledons, leaf disks, internodal sections, tuber discs, microtubers or wounded *in vitro* plantlets as the target tissues. A wide range of potato and tomato genotypes have been transformed with efficiencies ranging from 10% to 41% in tomato and 40%–100% in potato [11,13,25–27]. In addition, thanks to data flowing from genomic studies, novel tools for temporal and tissue-specific manipulation of gene expression in tomato fruits and in potato tubers have been produced and flexible gateway toolkits for targeted transgene expression and silencing in *Solanaceae* have been assembled [16,28–31].

Today it is possible to efficiently transform *Solanaceae* crops; however, one limitation of using food crops as bioreactors is the low-level of recombinant protein accumulation in these species. Nevertheless, several techniques are available that can be used to increase the level of protein expression in plant cells (several options to achieve overproduction of recombinant proteins were recently reviewed in [32]). Codon-optimization of the transgene, introduction of different regulatory elements and targeting of expressed proteins to special organelles are all factors that can enhance transcription and translational efficiencies and yield of recombinant proteins expressed in plant cells. For example, Jha *et al.* [33] demonstrated that targeting recombinant human α_1_-proteinase inhibitor to different subcellular compartments in transgenic tomato plants could influence final yield, biological activity and *in planta* stability of the recombinant protein. In addition, the level of transgene expression in plants can be affected by the activity and specificity of the promoters. Recently, there has been a vast amount of effort invested in discovering various types of promoters. For example, in tomato several fruit-specific promoters have been recently identified and utilized [28,34–36]. Targeting recombinant protein expression to the edible parts of the transgenic crop using fruit-specific promoters can be convenient for the production of oral plant-derived vaccines and to avoid non-specific alterations at whole plant levels. The identification of novel promoters expressed in specific tissues or during specific stages of development are proceeds of novel genomic resources and new high throughput sequencing methods such as RNA-Seq that allow evaluation of changes in expression of the transcriptome [9,32].

The accumulation of recombinant proteins in plant cells is dependent not only by the synthesis of the products but also on its degradation. Therefore, the potential of tomato cathepsin D inhibitor as a companion stabilizing agent for the protection of cytosol-targeted recombinant proteins in plant was recently investigated [37]. The authors demonstrated a proteome-wide, up-regulating effect of this inhibitor on endogenous leaf proteins in potato and a stabilizing effect *in planta* improving the accumulation of the cytosol-targeted heterologous protein human α_1_-antichymotrypsin.

An alternative to conventional nuclear transformation for the expression of high-yield recombinant protein in food crops is stable genetic transformation of plastids. The integration and expression of transgenes in the plastid genome presents several advantages including high and stable production levels of foreign protein attainable; precise integration into the host plastid genome that relies on homologous recombination; absence of epigenetic effects; reduced risk of introducing transgenes into the food-chain; and the possibility of co-expressing multiple transgenes from prokaryotic-like operons [38–40]. Previously, plastid transformation of species other than tobacco has been limited by low transformation frequencies and low transgene expression usually achieved in non-green plastid. However, today, protocols for stable plastid transformation in tomato and potato have been developed and have been made available to the scientific community [41–44]. The work of Valkov and colleagues [42] is noteworthy as they report an improvement in potato transformation efficiency at levels similar to those obtained for tobacco. This result was achieved by the modification of the selection/regeneration procedure and by using novel vectors containing potato-flanking sequences for transgene integration by homologous recombination in the plastome. In addition, regulatory sequences that could increase protein expression in tomato chromoplasts and potato amyloplasts have been identified and tested [42,45,46]. Caroca *et al.* [46] used tomato plastid transformation to test combination of promoters and 5′UTR for their potential to confer active gene expression in chromoplasts. The authors identified chimeric expression elements that trigger high-level protein accumulation in chromoplasts. The best-performing promoter-UTR combinations resulted in accumulation of the reporter protein GFP to up to 1% of total cellular protein of the ripe tomato fruit, which is comparable to the GFP accumulation level achievable in chloroplast of green leaves. Tomato transplastomic plants have been used successfully to produce nutraceutical and biopharmaceutical proteins [43,47,48]. For example, Apel *et al.* [47] introduced lycopene β-cyclase genes from the eubacterium *Erwinia herbicola* and daffodil (*Narcissus pseudonarcissus*) into the tomato plastid genome in order to enhance carotenoid biosynthesis inducing lycopene-to-β-carotene conversion.

Hairy root cultures present an alternative system for producing useful pharmacological compounds in crops [49]. In nature, hairy roots are a disease of plant tissues infected by the soil bacterium *Agrobacterium rhizogenes. A. rhizogenes* transfers specific genes (*rol* genes) from its endogenous root inducing plasmid (Ri plasmid) to alter the auxin/cytokinin perception of the host cells inducing neoplastic root and hair proliferation and growth [50]. Hairy roots provide a genetically stable transgenic tissue culture system that grows rapidly in simple phytohormone-free media. With respect to production of foreign proteins, hairy roots are easily bio-contained in a controlled *in vitro* environment; they can be scaled up to produce large amounts of biomass in industrial scale bio-reactors [51–53]; and have the potential for rhizosecretion [54]. Hairy roots of many different plant species have been utilised to produce various recombinant proteins at varying yields and there have been studies into producing recombinant proteins in hairy roots from crop plants such as tomato and potato. For example, Sunil Kumar *et al.* [55] optimized the generation and growth of potato hairy roots for the production of the Hepatitis B surface antigen (HBsAg). De Guzman *et al.* [56] reported production of the *E. coli* B-subunit heat labile toxin antigen in tomato hairy root cultures (~10 μg/g blotted weight, BW) and compared the productivity against hairy roots of tobacco (~100 μg/g BW) and petunia (~100 μg/g BW). While tomato yielded the least amount of antigen overall, the antigen accumulation was reasonable when compared to other plant systems producing LTB [57–59]. Unfortunately, oral delivery of vaccine antigens within hairy root tissue has proven less efficient than other plant tissues perhaps due to the cell walls being too thick and not releasing enough antigen from the cells at optimal locations throughout the digestive tract [60,61]. Secretion or purification of recombinant proteins from hairy roots may prove to be the more realistic approach for this platform.

## 3. Pharmaceutical Production in Food Crops

### 3.1. Fruit

Recently, tomato plants have been used as vehicles for the expression and oral delivery of vaccines since tomato can be ingested without prior treatment, generates abundant biomass at low cost, has flexible growth conditions, a short life cycle, contains the natural adjuvant α-tomatine, and is particularly rich in nutritional compounds such as carotenes and phenolics [2,14,15,62]. Fruit has been sometimes discarded as biofactories since they have a low protein content per fresh weight; however, transgenic tomato fruits can be easily freeze-dried, increasing the product yield and obtaining powdered material that can be made into batches with consistent antigen content and stored for long periods without the need for refrigeration.

Several studies reported the production of transgenic tomato plants for the expression of viral antigens, including rabies virus, foot-and-mouth disease virus, human papilloma virus, *Yersinia pestis* and for the production of therapeutic proteins (Table 1, [63]). Many investigations have focused on the development of novel mucosal vaccines against HIV and HBV (Hepatitis B virus) in transgenic tomato plants. These tomato-made vaccines are proposed to be inexpensive, heat stable and easy to administer. For example, Shchelkunov and colleagues [64] expressed in transgenic tomato plants the synthetic chimeric gene *TBI-HBS* encoding immunogenic ENV and GAG HIV-1 epitopes and the surface protein antigen (HBsAg) of hepatitis B virus and demonstrated that in mice oral administration of dried tomato tissues stimulated both serum and secretory HIV- and HBV-specific antibodies. Ramirez and colleagues [65] expressed the HIV-1 Tat protein in transgenic tomato fruits and demonstrated that the orally delivered tomato-based vaccine raised mucosal IgAs and induced serum IgGs with neutralizing activity in mice. More recently, Cueno *et al.* [66] demonstrated the preferential expression of a Tat-GUS fusion protein produced in tomato plants. Protein extracts intradermally injected into Balb/c mice were found to induce both humoral and cellular immune responses. In addition, Zhou *et al.* [48] expressed the HIV antigens p24 and Nef from the plastid genome of tomato. HIV antigen accumulation reached values of approximately 40% of total leaf protein and 2.5% of total protein in green tomatoes.

Bacterial antigens have also been expressed in transgenic tomato plants (Table 1). One example is the expression of a plant-optimized synthetic gene encoding the multiepitopic protein sDTP (diphtheria-pertussis-tetanus). The synthetic gene contained six DTP immunoprotective exotoxin epitopes and two adjuvants [78]. Recently, the same group demonstrated that in mice, three oral doses with freeze-dried material from the tomato-derived multicomponent vaccine elicited specific systemic and mucosal antibody responses [15]. Tomato-made vaccines have also been used as a novel strategy for the development of vaccines against cholera. Oral vaccines would be particularly suitable to protect against pathogens that infect through intestinal surfaces since this delivery route best induces a mucosal immune response [14,79]. Recent examples are the production in transgenic tomato plants of: (i) CTB (Cholera toxin B subunit), (ii) TCPA (toxin co-regulated pilus subunit A) of *Vibrio cholerae* and its immunogenic epitopes P4 or P6 fused to CTB and iii) ACFA (accessory colonization factor subunit A) of *Vibrio cholerae* and ACFA fused to CTB [67–69].

Tomato plants have been also used as platform for the production of therapeutic proteins. Recently Jha *et al.* [33] demonstrated the feasibility of using tomato plants for the production of stable, glycosylated and biologically active recombinant human α_1_-proteinase inhibitor. In addition, Kim *et al.* [34] reported the stable production of human β-secretase in transgenic ripe tomato fruits.

The capacity of fleshy fruits to accumulate functional antibody has been demonstrated by Juarez *et al.* [77]. The authors described the production of transgenic tomato plants expressing a recombinant human immunoglobulin A selected against the VP8* peptide of the model rotavirus strain SA11. Minimally processed fruit-derived products showed anti-VP8* binding activity and strongly inhibited virus infection in an *in vitro* virus neutralization assay. In addition, this paper dealt with the concerns often raised regarding possible contamination of the food chain with transgenic materials. In order to label the transgenic lines expressing the antibodies, sexual crossing were made with a transgenic tomato line expressing the genes encoding *Anthirrhinum majus Rosea1* and *Delila* transcription factors. These transcription factors ectopically activate anthocyanin biosynthesis in tomato fruit. The resulting purple-colored extracts from the transgenic fruit contained high levels of recombinant antirotavirus neutralizing human IgA in combination with increased amounts of health-promoting anthocyanins.

### 3.2. Tuber

Transgenic potato tubers as bioreactors offer advantages such as long-term storage tissue, abundant biomass, short growth cycle, high nutritional value and the high stability of recombinant proteins accumulated in the tubers during long period of storage [16,17]. Potato has been used in the last few years as a model system for the expression of bacterial, viral antigens and autoantigens (Table 2) and preliminary results from human clinical trial studies conducted with potato-based vaccines were promising [18,19,80]. In addition, mucosal immunity has been induced by orally administered transgenic potato plants [12,81–83]. Warzecha *et al.* [83] demonstrated that ingestion of transgenic tubers expressing the HPV11 L1 capsid protein activated anti-VLP immune responses that can be boosted by subsequent administration of purified insect cell-derived VLPs. Chen *et al.* [12] produced transgenic potato plants expressing GP5 protein of the porcine reproductive and respiratory syndrome virus (PRRSV) and showed that, in mice, oral administration of crude protein extracts from transgenic tubers elicited both serum and gut mucosal-specific antibodies.

Potato plants have also been used to produce several therapeutic proteins (Table 2). One example from Tremblay and colleagues [84] demonstrated high-yield of soybean agglutinin (SBA), a specific *N*-acetylgalactosamine-binding plant lectin, in potato tubers. The recombinant SBA retained its ability to induce hemagglutination, was similarly glycosylated to the native SBA and retained its binding specificity for *N*-acetylgalactosamine. In addition, the recombinant SBA was highly resistant to degradation in simulated gastric and intestinal fluid.

One disadvantage of using transgenic potato for the production of antigenic proteins is the poor expression of the foreign proteins. In this regard, it is of interest to mention the work from Youm *et al.* [89] who examined the antibody response in mice orally immunized using various doses of potato-derived major surface antigen of hepatitis B virus (ranging from 0.02 to 30 μg potato-derived antigen). Approaches to increase the yield of recombinant protein expressed in transgenic plants include down regulation of native proteins within the tubers and targeting the protein to the cell secretory pathway. Tremblay *et al.* [17] investigated whether minor interruption of starch biosynthesis can have a positive effect on tuber protein content and on tuber biomass. In order to increase the efficiency of the crop potato as a bioreactor, they used an RNAi approach to knock down ATP/ADP transporter in *Solanum tuberosum.* The authors identified a new line (riAATP1-10) with reduced starch accumulation, increased biomass yield and increased total protein levels. The potential of this line as a new bioreactor candidate was tested by expressing a human single-chain variable fragment (scFv) antibody. Protein expression in the riAATP1-10 line translated into a nearly 4-fold increase in product yield. Kim *et al.* [90] also utilized the RNAi technology to knockdown various patatin isoforms in potato tubers for the development of a more efficient protein expression system.

In an attempt to improve the yield, the activity and the stability of recombinant protein, Badri *et al.* [91,92] used protein targeting to produce transgenic potato lines expressing the protein bovine aprotinin targeted to the cytosol, the endoplasmic reticulum (ER) and the apoplast. Using a novel SELDI-TOF MS (Surface-enhanced laser desorption ionization time-of-flight mass spectrometry) procedure, the authors were able to demonstrate that the recombinant protein targeted to the ER showed good accumulation levels, however, was processed in the ER compartment of plant cells [91]. In a subsequent paper, the authors used a combination of SELDI TOF MS and 2-D gel analyses to demonstrate that aprotinin retention in the ER was associated with a decrease of leaf soluble protein content and down-regulation of proteins implicated in protein synthesis and maturation. This suggests unintended metabolic interference in transgenic plants [92]. These data demonstrate the importance in plant-made vaccine design of considering also the possible effects of the foreign protein expression on native protein accumulation and endogenous metabolism of the host plant factory.

Additional factors that could limit recombinant protein expression include processes such as silencing, premature polyadenylation, mRNA stability, and improper codon usage. Mathew *et al.* [93] showed the importance of eliminating spurious polyadenylation signals within the coding sequence of the Narita 104 virus capsid protein. Such an exercise increased foreign protein expression in potato plants. Finally, the variability in antigen expression could depend also on the different plant growth conditions used. For example, Mikschofsky *et al.* [94] compared greenhouse and field production of potato-made foreign protein using potato expressing VP60 (structural capsid protein of the rabbit hemorrhagic disease virus), CTB and the marker protein NPTII and concluded that equal or higher expression levels with lower variability of foreign protein could be expected in the fields compared to greenhouse production.

## 4. Issues Remaining for Plant-Made Pharmaceuticals

### 4.1. Business Development

While the USA has certain state and local entities, and government and health entities such as NIH and NIAID fortunately funding multimillion dollar developments in the plant-made vaccine field, today’s economic climate has created a hesitancy in the rest of the world for support of a comparatively new come recombinant protein production platform, particularly from industry/big pharmaceutical companies. According to a survey performed by BioPlan of biopharmaceutical manufacturers, only 14% of respondents plan to increase manufacturing using plant expression systems in the next five years with most companies continuing to focus on traditional mammalian cell, bacterial, and yeast expression technologies [95].

It is presently thought that a knowledge gap between existing technology skills and experience and those required for plant technologies, particularly in production and downstream processing lays to blame for the lack of wider adoption of plant-made pharmaceutical technology. Batch processing technology used in conjunction with techniques that increase product yield (e.g., using deconstructed viral vectors, inducible promoters, secretion systems and streamlined purification) have relieved the concerns about variable and low antigen expression. We therefore focus on the remaining issues slowing adoption of plant-made pharmaceuticals.

### 4.2. Knowledge Gap

The lack of know-how to economically produce and upstream process technologies arising from a plant-made vaccine laboratory and turn them into a business venture might be rapidly solved through industry involvement. However, industry’s involvement is all too infrequent and strategies need to be put in place to obtain the required expertise/experience from other sources. With the tomato and potato industries easily producing crops of commercial size, the problem lies in growing these crops then processing them at commercial scale in a manner that conforms to the appropriate country regulations for Genetically Modified Organisms (GMOs) and pharmaceutical production. Perhaps what is required is, after learning the current agricultural practices in producing tomatoes and potatoes, further uptake of programs such as that started at Stanford. Stanford scientists and engineers are being sent into the “startup community” by the federally funded Innovation Corps [96] to learn how to translate their research into a business. These teams include a plant-made vaccine team at UC Davis lead by Karen McDonald that is developing a way to produce vaccines and other medical treatments faster than the current methods by using tobacco plants [96]. In the case of plant-made vaccines in tomatoes and potatoes, biotechnologists would need to consult with tomato and/or potato food technology experts in conjunction with pharmaceutical Good Manufacturing and regulatory experts to determine how the downstream processing could occur economically to scale and to meet the regulations in place.

Not surprisingly, when industry has invested in plant-made pharmaceutical technology, the field has rapidly progressed. Dow AgroSciences collaboration with Arizona State University and Benchmark Biolabs resulted in the world’s first licensing of a plant-made vaccine, New Castles Disease Virus Vaccine. Pfizer’s later stage investment in Protalix’s plant cell-made Gaucher’s therapeutic (Elelyso) has seen the therapeutic receive approval from the USA’s FDA for use in humans (May 2012) at a cost 25% below that of competing products [97]. It is hoped that these successful ventures into the plant-made recombinant protein world will assist seeing production of pharmaceutical proteins in plants as a feasible investment for major pharmaceutical companies.

### 4.3. Oral Delivery

Oral delivery is an economically and physically efficient way to administer pharmaceutical proteins to animals and humans. Among the obvious benefits such as ease of delivery and eliminating the need for needles, oral delivery can enable simultaneous stimulation of antigen-specific mucosal, humoral, cell mediated and systemic immune responses. Plant-made subunit vaccines that will likely be feasible for oral delivery include antigens that aggregate in forms that are recognized at specific mucosal sites where immune responses are triggered. These include virus like particles (VLPs) and multimeric bacterial toxins.

One advantage of mucosally delivered plant-made vaccines is the presence in several plants species of compounds, such as lectins and saponins, which have shown strong adjuvant activities [98]. Such compounds could serve as natural adjuvant potentiating mucosal immune responses. In this regards, it is of interest that solanaceous crops are rich in steroidal saponins such as α-solanine and α-chaconine in potato and α-tomatine in green tomato fruits and leaves. A molecular aggregate formulation (*Tomatine*), which was based on α-tomatine, was reported to be capable of stimulating antigen-specific humoral and cellular immune responses that contributed to protection against malaria, *Francisella tularensis* and regression of experimental tumors [98–100].

On a number of occasions, plant-made vaccines have shown to be able to induce antigen-specific immune responses when orally administered in animal and human studies [57,59,101,102]. However, orally delivered, subunit vaccines have not yet achieved commercial success due to variability of the induced immune responses. This is most likely caused by varying degradation of the antigens in the complex and dynamic environment of the gastrointestinal tract (GIT). Consequently, the trend in plant-made vaccine research has been to use plants merely as the production system before the product is purified, characterised and eventually delivered parenterally [103,104]. In order for oral delivery to become a more attractive delivery system, more research is required into stabilizing and/or protecting vaccine antigens from premature degradation within the gastrointestinal environment and targeting specific areas of the immune system for more effective responses.

Encapsulation of the immunogen within the plant cell matrix during administration may provide some protection from degradation by gastric enzymes and acids. Experiments performed by Pelosi *et al.* [60] attempted to elucidate the optimal conditions for oral delivery of plant-made vaccines by delivering recombinant LTB produced in various plant tissue types, in different formulations, to mice. They also investigated the site of release of the antigen and the corresponding induced immune response in the mouse gut. Their results showed that encapsulation of LTB within the plant cell provides some protection of the antigen throughout the mouse GIT, however the cell type also plays a role in determining the site of antigen release. In this study, the fibrous root tissues were shown to not release the majority of the antigen contained within its cells in comparison to the tomato fruit that released the antigen early along the digestive tract [60]. Formulation of the delivered vaccine also affected the induced immune responses where antigen delivered in a lipid based formulation resulted in more robust immune responses than when delivered in aqueous formulation. In another example, Zhang *et al.* [100] evaluated tomato fruit and potato tuber as a vehicle for the production and oral delivery of recombinant Norwalk virus capsid protein. The authors showed that freeze-dried or air-dried tomato fruits were highly immunogenic when administer orally in mice whereas the same strategy was less successful using potato tubers, thanks also to the presence of α-tomatine in the tomato-made vaccine [100]. In addition, air-dried tomato fruits could induce more robust immune responses than freeze-dried powder probably because the plant cell matrix and membrane system were conserved by air-drying.

Therefore, it is important to consider cell type, formulation and site of antigen release in future studies of orally delivered antigen and resulting immunogenicity, particularly with regards to induction of systemic immune responses after oral delivery. Although oral delivery of plant-made vaccines continues to be investigated, the field has only begun to start to unravel and further studies are required.

### 4.4. Downstream Processing

Biotechnological production systems are often separated into early-stage, upstream and downstream processing. The early-stage processes involve vector construction, host transformation, initial expression analyses and process optimization to result in product production. The upstream process extends from selecting an elite line for sequential scale-up tests to when the crude material containing the product is harvested. Finally, the downstream processing focuses on treatment (extraction and purification) of the harvested material to obtain the product in a suitable form and quality that complies with regulations, and finishes with the manufacturing of a bulk product ready for final testing. Each of these phases require extensive time and funding and without careful planning the process may come to a stop at any particular phase. This section will address the downstream processes in more detail.

A protein’s function often relies on its tertiary structure that enables binding of the protein to other molecules or proteins. Effective extraction and purification of intact functional proteins from organisms or biological tissues can be a laborious and an expensive process, especially extraction from plant tissues (due to the abundance of proteases and other interfering compounds such as the cell walls, polysaccharides, and various secondary metabolites such as phenolic compounds) [56,105]. This stage often accounts for the majority of the cost of plant-made pharmaceutical production and needs to be addressed if plant-made pharmaceuticals continue to be purified. The nature of the antigen itself plays a major role in determining the modes of extraction and time of harvest. Examples include the antigen stability at different temperatures and pH impacting product quality and yield, and whether or not it requires an added detergent for membrane dispersal due to the protein containing a transmembrane domain. While the pH of potato tubers does not vary all that greatly, ranging from 5.5 to 6.2 in cultivars and down to 5 in wild species [106], the pH in tomato fruits changes as they ripen dropping from 6 in fully sized green fruit to 4 in mature red fruit (personal data and [107]). It is therefore prudent to be aware of the optimal pH for quality and yield of the antigen of interest and harvesting the fruit when the pH is optimal.

The next series of steps involve removal of bulk waste plant material by filtration and/or centrifugation, and finishing with more complex purification procedures, including immunoprecipitation and/or chromatography. Extraction and purification of a desired product from plant tissues require careful consideration and tailoring of the processes to the specific product being purified. Before being administered to animals, consideration of the original plant material may be required due to the fact that some plant types contain harmful alkaloids and toxins that must be removed (e.g., nicotine in tobacco and cyanide in crop cassava). Fortunately, the *Solanaceae* food crops do not have this issue. An increase in research into purification strategies has given rise to various techniques for purifying specific proteins derived from particular host production platforms. For example, Azzoni *et al.* [108] reported recovery of 49% of aprotinin (a protease inhibitor) extracted from transgenic maize using a trypsin-agarose column and purification of recombinant proteins from seeds using oleosin partitioning [109] or ELP-Intein Coupling System in rice grains [110] were also described.

Another technique used to ease purification was shown by Lin *et al.* [111] where they fused their gene of interest (*sgp130*–used to treat rheumatoid arthritis and colon cancer) to repeated sequences of the elastin-like protein (ELP) in a process called inversion transition cycling. This process utilizes the ability of ELP-fusion proteins to become insoluble in a raised temperature environment and lead to an accumulation of 141 μg/g FW of intact sgp130 protein that could then be easily purified by separation. The resulting purified sgp130 protein was shown to be functionally active.

Some plant-made biopharmaceuticals, such as topically applied products and products secreted into the media by cell or tissue cultures, may require only minimal purification and thus reduce the time and labor required to purify. Affinity chromatography, ion exchange techniques and gel filtration/separation may be used to concentrate and further purify these products. An example of the ease of purification of a secreted product was shown by Drake *et al.* [112] whereby they produced the monoclonal antibody Guy’s 13 in tobacco plants grown in a hydroponic system. They were able to detect up to 58 μg/g root dry weight/day of Guy’s 13 that was secreted into the hydroponic medium, and using affinity chromatography were able to recover ≥ 90% of purified functional product. Since tomato plants grow well in hydroponic systems, this possibility of minimal purification of proteins within hydroponic medium should be tested.

While we presented above a number of possible means to downstream process pharmaceuticals from plant materials, these too are still applied at a laboratory scale and need to be economically scaled up to batch production intensity.

### 4.5. Regulations

Plant-made pharmaceuticals will be subjected to the same quality control and safety standards as other pharmaceuticals derived from bacterial, yeast/fungi, animal or mammalian cell systems. However, a main concern regarding the production of pharmaceuticals in plants is the safety of the food chain and natural ecosystems from contamination with pharmaceuticals. This could occur if: pollen from transgenic plants out-crossed with food crops or wild plants; equipment used in the processing of the plant-made pharmaceuticals was used without thorough decontamination; and/or if fields used for growing transgenic crops were subsequently used to grow normal food crops without a pre-assigned decontamination period. This is of concern especially if agricultural crops, such as corn, rice, potatoes and tomatoes, are used as platform for the production of recombinant proteins. Therefore, containment of the transgene is of the utmost importance and can only be achieved by stringent regulations regarding geographical isolation, and physical containment in glasshouses. Indoor growing could eliminate not only the concerns regarding mixing transgenic and non transgenic foodstuffs, but also contamination with other materials such as insect parts, bird excrements and pesticide drifts from other crops [113]. Greenhouses could increase the cost of the final products but could enable year-round production and make cultivation conditions controlled and adjustable.

It is perceived that there is a lack of regulatory framework concerning plant-made vaccines and this has been offered as one reason why development of this technology beyond proof-of-concept studies has been slow. However, the successful licensure of a plant-made Newcastle Disease Virus vaccine for poultry by Dow AgroSciences does at least demonstrate that plant cell cultures can be regulated for animal health vaccines under the existing regulations of the USDA. In fact there is a growing number of plant-made vaccine and therapeutics navigating their way through the regulatory systems of different countries (Table 3) and these activities are adding to the clinical data available on effectiveness of plant-made therapeutics. The probability of harvesting every transgenic potato tuber in a field is low and therefore raises concern due to the possibility of gene escape or food chain contamination. However, this would be circumvented through maintenance of specific fields and farming machinery for transgenic potato production (isolation). Production of potatoes in contained conditions (greenhouses) is not optimal but possible while greenhouse production of tomatoes is already a commercial technique with approximately 1000 acres in USA in 2011 [114]. It appears that while the regulatory pathway is complex and time consuming, with some foresight it is not significantly more so with plant-made vaccines as compared to other recombinant therapeutics/vaccines.

## 5. Conclusions

In this review, we provided an update on the use of food-crops for the production of pharmaceuticals and also we described the novel biotechnological tools available today for the economically important *Solanaceae* family. As a result of the efforts of researchers worldwide, the development of transgenic edible crops as bioreactors has made considerable advancements, providing plant biotechnologists with many new tools to solve issues, such as the knowledge of promoter and sequences, and the level of gene expression and the maintenance of a stable steady-state level of plant-made foreign protein. Researchers are now able to choose the most suitable host plant species, sub-cellular compartments/organelles (e.g., plastids) and the different plant organs in which to produce their desired recombinant protein [40]. These choices can influence not only the accumulation level obtained in the transgenic material but also the immunogenicity of the recombinant protein produced. The immunogenicity of the plant-made vaccines can be influenced also by the formulations used for the delivery of the plant-made pharmaceutical protein [100].

Today we know that is possible to produce biologically active and immunogenic protein in *Solanaceae* food crops; however, now more studies need to focus on optimizing delivery of plant-made proteins in order not to lose the claimed advantages of this system including increased safety, low production cost and ease of delivery.

## Figures and Tables

**Table 1:** Examples of recombinant proteins produced in *Solanum lycopersicum* in the last five years (2007–2012).

Foreign protein	Source of transgene	Production level	Protein functionality	Reference
CTB-P4; CTB-P6 and TCPA	*Vibrio cholerae*	0.17%; 0.096% and 0.12% TSP fruit	Assembly of pentameric chimeric proteins	[67]
CTB	*Vibrio cholerae*	0.081% TSP fruit	Immunogenic in mice	[68]
ACFA and ACFA-CTB	*Vibrio cholerae*	0.25% and 0.08% TSP fruit	NR	[69]
p24-Nef	HIV-1	40% TSP leaves, 2.5% TSP green fruits	NR	[48]
Tat-GUS	HIV-1	2–4 μg/mg plant protein	Immunogenic in mice	[66]
Tat	HIV-1	1 μg/mg fruit (dry weight)	Immunogenic in mice	[65]
PRS-S1S2S	HBV	0.02% TSP fruit	Assemble into capsomers and VLP	[70]
Nucleoprotein N	Rabies virus	1%–5% TSP fruit	Immunogenic in mice	[71]
sDPT	*Corynebacterim diphtheria*, *Bordetella pertussis*, *Clostridium tetani*	20 Lf/g; 88 Lf/g and 270 ng/g freeze-dried fruit	Immunogenic in mice	[15]
L1-E6/E7	HPV 16	0.1% TSP fruit	Immunogenic in mice	[72]
P1-2A3C	FMDV	NR	Immunogenic in guinea pigs	[73]
β-amyloid	human	80–58 ng/mL extracts	Immunogenic in mice	[74]
α_1_-antitrypsin	human	1.55% TSP leaf	Biologically active	[75]
EGF	human	3.48 ng/g fruit	Protect mice against alcohol-induced gastric injury	[76]
BACE1	human	136 ± 7 ng/mg TSP fruit	Biologically active	[34]
α_1_-PI	human	1.5%–3.2% TSP leaves	Biologically active	[33]
hIgA_2A1	human	3.6% ± 0.8% TSP fruit	Neutralize rotavirus infection *in vitro*	[77]

NR: Not Reported; PRS-S1S2S: HBV large surface antigen gene; L1-E6/E7: chimeric particle containing the L1 sequence and a string of T-cell epitopes from E6 and E7; P1-2A3C: structural polyprotein P1-2A and protease 3C from foot-and-mouth disease virus; BACE1: β-Site APP cleaving enzyme 1; α-PI: α-proteinase inhibitor; hIgA_2A1: human immunoglobulin A selected against the VP8* peptide of rotavirus SA11 strain.

**Table 2:** Examples of recombinant proteins produced in *Solanum tuberosum* in the last five years (2007–2012).

Foreign Protein	Source of transgene	Production level	Protein functionality	Reference
GP5	PRRSV	4.7 μg/g leaves, 1.2 μg/g tubers	Immunogenic in mice	[12]
CTB-VP1	Foot and mouth disease virus	0.1%–0.13% TSP tuber	NR	[85]
FimA	*Porphyromonas gingivalis*	0.03% TSP tuber	NR	[19]
INS-RTB	human	NR	Biologically active	[86]
SBA	soybean	0.3% TSP tuber	Retained authentic SBA activity	[84]
IFN-α1	*Salmo salar*	NR	Antiviral activity	[87]
staphylokinase	bacteria	NR	Biological activity	[88]

NR: Not Reported; FimA: fimbrial protein, INS: proinsulin.

**Table 3:** Milestones in the development of plant-made pharmaceuticals.

Year	Event
1986	Conception of plant-made vaccine strategy by Curtiss and Cardineau
1988	First plant expression data of vaccine antigen, patent filed (US patents issued in 1997)
1990	First animal immunogenicity data for plant-made antigen. Patent application published by WIPO
1997	First human clinical trial with plant-made vaccine, phase I (potato LTB)
1998	First human clinical trial with plant-made antibody, phase 1 (purified, against dental caries)
2003	Sixth human clinical trial with plant-made vaccine, phase I (corn LTB)
2005	Development of Magnifection and corresponding increase of recombinant protein expression to commercially viable levels
2006	First plant-made vaccine licensed for use in animals (NT1 cells NDV vaccine)
2006	Phase I/II trials investigating a purified, plant-made therapeutic for Gaucher’s disease (carrot cell suspension)
2008	Demonstration of plants as rapid, vaccine production systems (purified injectable for influenza)
2008	First human clinical trial with a plant-made vaccine against cancer (antibody)
2009	First Phase III clinical trial investigating plant-made therapeutic for Gaucher’s disease (sponsored by Proalix)
2010	First Phase II trial with plant-made antibody against tooth decay (CaroRX)
2010	Expanded access granted for carrot-made treatment for Gaucher’s disease
2012	FDA permission granted for use of carrot-made treatment for Gaucher’s disease in humans

WIPO, World Intellectual Property Organisation; HBsAg, Hepatitis B surface antigen; LTB, *Escherichia coli* heat labile enterotoxin B subunit; NDV, Newcastle Disease Virus; NT1 Cells, plant cell culture of *Nicotiana tabacum*; NVCP, Norwalk virus capsid protein; RVgp, Rabies virus glycoprotein.
